# Inhibition of the activin receptor signaling pathway: A novel intervention against osteosarcoma

**DOI:** 10.1002/cam4.3581

**Published:** 2020-11-12

**Authors:** Daniela Meier, Andreas Lodberg, Ana Gvozdenovic, Giovanni Pellegrini, Olga Neklyudova, Walter Born, Bruno Fuchs, Marco Eijken, Sander M. Botter

**Affiliations:** ^1^ Department of Orthopedics Balgrist University Hospital Zurich Switzerland; ^2^ Department of Biomedicine Aarhus University Aarhus Denmark; ^3^ Department of Pulmonary Medicine Aarhus University Hospital Aarhus Denmark; ^4^ Laboratory for Animal Model Pathology Institute of Veterinary Pathology, University of Zurich Zurich Switzerland; ^5^ Department of Renal Medicine Aarhus University Hospital Aarhus Denmark; ^6^ Department of Clinical Immunology Aarhus University Hospital Aarhus Denmark

**Keywords:** activin type II receptor, follistatin, osteosarcoma, pathological bone remodeling, zoledronic acid

## Abstract

Osteosarcoma is a cancer of pathological bone remodeling with high mortality and severe comorbidity. New therapies are urgently needed. Activin A, a member of the transforming growth factor β (TGFβ) superfamily, has been suggested to stimulate proliferation and invasion of osteosarcoma cells in vitro, thus representing a potential therapeutic target. In this study, inhibition of the activin receptor signaling pathway was explored as a therapy for osteosarcoma. In a murine intratibial osteosarcoma xenograft model, two types of inhibitors were tested: (a) a soluble activin type IIA decoy receptor (ActRIIA‐mFc), or (b) a modified variant of follistatin (FST_ΔHBS_‐hFc), either alone or in combination with a bisphosphonate. Both inhibitors reduced primary tumor development by nearly 50% compared to vehicle treatment. When ActRIIA‐mFc was combined with bisphosphonate, the effect on tumor size became even more pronounced (78% reduction vs. vehicle). Moreover, FST_ΔHBS_‐hFc increased body weight in the face of tumor progression (14% increase vs. vehicle), and ActRIIA‐mFc reduced the number of lung metastases when combined with bisphosphonate. The present study demonstrates a novel approach to treating osteosarcoma and encourages further investigation of inhibition of the activin receptor signaling pathway as an intervention against the disease.

## Graphical abstract

Inhibition of the activin receptor signaling pathway reduces tumor volume, pathological bone remodeling, and counteracts the cachexia that follows in the wake of osteosarcoma.

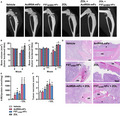

## 1 INTRODUCTION

Osteosarcoma is the most common malignant primary tumor of bone and it has a strong propensity for metastasizing to the lungs. In spite of multi‐agent chemotherapy, the 5‐year survival rate remain as low as a few percentages in the elderly (60+ years of age) and amounts to 30% in young patients (0–24 years of age), when distant disease is present at the time of diagnosis.^1^

Osteosarcoma is a cancer of pathological bone remodeling that includes a number of subtypes, differing in their anatomical location, histology, phenotype, or genetic profile.^2^ Consequently, tumor heterogeneity presents a challenge in the discovery of new therapies. To overcome this problem, targeting common factors for all subtypes may serve as a strategy. Selective blockade of the ubiquitous transforming growth factor β (TGFβ) superfamily represents such an option.

Activin A and growth differentiation factor 11 (GDF11) are members of the TGFβ superfamily that have recently been proposed as negative regulators of bone mass.^3^, ^4^, ^5^ Their neutralization have been reported to increase bone mass, bone strength, or bone formation markers in postmenopausal women or preclinical models of bone loss.^5^, ^6^, ^7^, ^8^ In addition, aberrant activin A signaling has been shown to invoke local and systemic inflammation and to be a driving factor in various cancers.^9^, ^10^

In osteosarcoma, pathological bone remodeling is thought to maintain a “vicious cycle” that nourishes tumor cells by release of growth factors stored within the bone matrix.^11^ This concept extends from Paget's “seed and soil” theory, originally formulated in relation to breast cancer.^12^ Neutralization of activin A and GDF11 in animal models of breast cancer or multiple myeloma has shown inhibition of tumor growth and a reduction in the number of bone metastases and osteolytic lesions.^13^, ^14^ In relation to osteosarcoma, a single in vitro study found activin A enhanced proliferation and invasion and migration of osteosarcoma cell lines.^15^ Activin A or GDF11 would, therefore, appear ideal targets, and yet, their modulation has never been attempted in in vivo models of osteosarcoma.

Activin A and GDF11 initiate signaling *via* membrane proteins known as activin type II receptors (ActRIIs; ActRIIA and ActRIIB). Collectively, compounds that prevent transduction through these receptors may be referred to as inhibitors of the activin receptor signaling pathway (IASPs).^6^, ^16^

Bisphosphonates are chemically stable derivatives of inorganic pyrophosphate that have a natural predilection for hydroxyapatite crystals.^17^ During bone remodeling, bisphosphonate is absorbed by the osteoclast and incorporated into the cell's adenosine triphosphate production.^18^ This sabotage of the energy pool is cytotoxic and ultimately leads to the death of the osteoclast. Because of the antiresorptive capability bisphosphonate is a common therapeutic agent in disease with osteoclast mediated bone loss, including some forms of metastatic malignancy.^19^ Studies of osteosarcoma cell lines suggest bisphosphonate is also toxic to the osteosarcoma cell type, and in murine models of osteosarcoma bisphosphonate improves survival and reduces the number of bone lesions.^20^, ^21^, ^22^

In this study, we explored inhibition of the activin receptor signaling pathway as a therapy for osteosarcoma. Two types of IASPs were tested: 1) a soluble activin type IIA decoy receptor (ActRIIA‐mFc),^4^ and 2) a modified variant of the native antagonist, follistatin (FST), (FST_ΔHBS_‐hFc).^16^ The two IASPs were tested alone or in combination with bisphosphonate in an intratibial human xenograft osteosarcoma mouse model. The purpose of including bisphosphonate was to investigate the combination of an established antiresorptive with an inhibitor of growth factors that are likely released by the destruction of bone matrix as is suggested by the “seed and soil” theory.

Both IASPs were found to reduce tumor development and tumor‐associated bone remodeling with the greatest effect observed in combination with bisphosphonate. Inhibition of the activin receptor signaling pathway represents a novel therapeutic strategy against osteosarcoma.

## 2 MATERIALS AND METHODS

### 2.1 Construction and expression and purification of ActRIIA‐mFc and FST_ΔHBS_‐hFc

The construction and expression and purification procedures of ActRIIA‐mFc and FST_ΔHBS_‐hFc were recently described in detail.^16^ Briefly, ActRIIA‐mFc represents a fusion protein of the human ActRIIA ectodomain fused to the Fc region of murine IgG2a, including a TGGG linker.

The FST analog, FST_ΔHBS_‐hFc, was generated by replacing the heparin‐binding sequence (HBS) of native FST_315_ by a structurally related sequence, so as to reduce its heparan sulfate binding capacity and thus increase half‐life.^23^ Finally, the protein was fused to the Fc of human IgG1.

The vehicle for both recombinant proteins was a buffer containing 50 mmol/L KPO_4_, 165 mmol/L sucrose, 0.01% Tween 20, adjusted to a pH of 7.4.

### 2.2 In vitro validation of therapeutics

The ability of ActRIIA‐mFc or FST_ΔHBS_‐mFc to prevent induction of Smad 2/3 signaling was quantified and compared to native FST_315_ in a bioassay of HEK293 cells containing a (CAGA)12‐luciferase sequence (a readout for Smad 2/3 signaling) as previously described.^16^, ^24^ Briefly, cells were stimulated with activin A and co‐treated with a concentration range of native FST_315_, FST_ΔHBS_‐mFc, or ActRIIA‐mFc. For the neutralization bioassay, the murine ortholog of FST_ΔHBS_‐Fc was used. Cells were lysed after 24 h and the luciferase signal was quantified. A dose‐response curve was plotted and the half‐maximal inhibitory concentration (IC_50_) calculated. The bioassay was performed four times using four different batches of recombinant protein.

The ability of ActRIIA‐Fc or FST_ΔHBS_‐hFc to inhibit activin A‐stimulated osteoclast formation was quantified in an assay utilizing peripheral blood mononuclear cells (PBMC) obtained from whole blood of healthy individuals and sorted for CD14 positive PBMCs with a human CD14 Selection Kit (#18058, STEMCELL Technologies). The manufacturers' instruction was followed and the Manual EasySep protocol with the Purple EasySep Magnet (#18000, STEMCELL Technologies) was used. Cells were cultured in MEM Alpha Medium supplemented with 15% heat inactivated fetal bovine serum. CD14 positive cells (60.000 cells per/well in a 96 wells plate) were differentiated in the presence of macrophage‐colony stimulating factor (m‐CSF, 25 ng/ml) and receptor activator of NFκβ ligand (RANKL, 60 ng/ml) as described by Susa et al.^25^ From day four, activin A (0.5 nmol/L), native FST_315_ (100 nmol/L), FST_ΔHBS_‐hFc (100 nmol/L), ActRIIA‐mFc (100 nmol/L), or the bisphosphonate, ZOL (0.5 µmol/L), were added to the tissue culture medium.

Following fixation, tartrate resistant acid phosphatase (TRAP) histochemistry was performed. Cells were incubated with 0.2 M acetate buffer for 20 min at room temperature, then, incubated with 0.5 mg/ml Naphthol AS‐MX phosphate (#855, Sigma‐Aldrich) and 1.1 mg/mL Fast Red TR Salt (#F8764, Sigma‐Aldrich) in the presence of 50 mmol/L sodium tartrate for 1 h at 37°C, and then, washed with PBS and stained with Hoechst (#H3570, Life Technologies, 1:10.000, 10 min, room temperature). Osteoclast studies were performed two times and in triplicate. Wells were photographed in quadruplicate and the number of TRAP positive, large multinucleated osteoclasts (approximately 100–200 µm) was determined after 14 days of culture using ImageJ (http://rsb.info.nih.gov/ij/).

### 2.3 Animals and osteosarcoma model

Human 143B cells (RRID: CVCL_2270) were obtained from the American Type Culture Collection (ATCC CRL‐8303). Cells were cultured in DMEM (4.5 g/L glucose)/HamF12 (1:1) tissue culture medium (Invitrogen), supplemented with 10% heat‐inactivated FCS (GIBCO), at 37°C in a humidified atmosphere (5% CO_2_). 143B cells were transduced with *lacZ* and *mCherry* gene constructs as previously described,^26^ resulting in the 143B/lacZ/mCherry cell line. Cells were authenticated by multiplex PCR (Microsynth) using the PowerPlex®16HS system (Promega) and verified by comparison with the database at the Collection of Microorganisms and Cell Cultures (DSMZ). Absence of mycoplasma was confirmed by PCR (results not shown).

The present study employed an intratibial human xenograft osteosarcoma mouse model together with 4 weeks of intervention therapy. Severe combined immunodeficient (SCID) mice, CB17/lcr‐Prkdc^scid^d/lcrlcoCrl, were obtained from Charles River Laboratories and kept for a 10‐day acclimatization period before orthotopic tumor cell injection. Orthotopic tumor cell delivery was *via* left intratibial injection with 10^5^ 143B/*lacZ*/*mCherry* cells as previously described.^27^ Following tumor growth, mice were treated with buprenorphine in accordance with current specifications from the local veterinary office.

### 2.4 In vivo imaging system

In vivo fluorescence imaging was performed for the visualization of *mCherry* expressing 143B cells within intratibial primary tumors. Tumor viability was assessed 1 week after tumor cell injection and thereafter regularly throughout the study using the IVIS Lumina XR (PerkinElmer) under gas anesthesia (2–5% isoflurane/O_2_). Epi‐fluorescence and spectral unmixing (excitation 605 nm, emission collected at 660, 680, and 700 nm) was analyzed using Living Image v 4.4 software (PerkinElmer) and presented as average radiant efficiency [(p/s/cm^2^/sr)/(µW/cm^2^].

### 2.5 Monotherapy against osteosarcoma

Forty‐eight 11‐week‐old female SCID mice were randomized into three groups according to weight: vehicle (KPO_4_, *n* = 12), ActRIIA‐mFc (*n* = 12), and FST_ΔHBS_‐hFc (*n* = 12). A fourth group, receiving standard antiresorptive therapy, bisphosphonate (ZOL, *n* = 12), was included for comparison. Treatment began 11 days after tumor cell injection. Both ActRIIA‐mFc (10 mg/kg) and FST_ΔHBS_‐hFc (10 mg/kg) were administered intraperitoneally, twice weekly. ZOL (100 µg/kg) was administered subcutaneously, twice weekly. During the experiment, three mice died prematurely, one in each of the active treatment groups; two mice were found dead in their cage, one mouse died during blood sampling.

Mice were weighed weekly and at sacrifice, 34 days after tumor cell injection. Tumor growth was monitored by caliper measurements and the Faxitron MX‐20 cabinet X‐ray system (Faxitron X‐ray LLC) as described.^27^ Lungs were perfused in situ and following excision micro and macro lung surface metastases were counted as described.^28^ The tibia and tumor tissue were dissected and stored in 4% of paraformaldehyde/PBS until analysis.

### 2.6 Combination therapy against osteosarcoma

Seventy‐two 11‐week‐old female SCID mice were randomized into six groups according to weight: vehicle (KPO_4,_ *n* = 13), ActRIIA‐mFc (*n* = 11), FST_ΔHBS_‐hFc (*n* = 12), ZOL (*n* = 12), ActRIIA‐mFc +ZOL (*n* = 12), and FST_ΔHBS_‐hFc +ZOL (*n* = 12). Three mice, two from the vehicle and one from the FST_ΔHBS_‐hFc group, were excluded because they did not develop a tumor, based on the absence of mCherry signal and lacZ staining following sacrifice. Two mice died during the in vivo µCT scan (procedure see below) due to respiratory insufficiency not related to treatment, one in the ActRIIA‐mFc and one in the ZOL group. Treatment began 11 days after tumor cell injection. The same dosage and injection protocol were applied as in the monotherapy experiment.

### 2.7 In vivo microcomputed tomography analysis

Animals were anesthetized (2–5% isoflurane/O_2_) and placed in the supine position while hind limbs were scanned with an isotropic resolution of 17 µm in a Bruker/SkyScan‐1176 in vivo X‐ray µCT (Bruker). Adhering to current guidelines,^29^ the following settings were used: X‐ray voltage 50 kV, tube current 500 µA, exposure 290 ms, frame averaging 2, with a rotation step of 0.8 degrees over a 198 degree trajectory (“180‐degree” setting), creating 247 raw image files per scan. Three‐dimensional datasets were generated using Cone‐Beam reconstruction software (NRecon v 1.6.9.18, Bruker).

Increasing soft tissue as a result of tumor expansion confounds the bone volume fraction (defined as bone volume (BV)/tissue volume), and thus, total BV was chosen as the primary µCT outcome. A region of interest of the tibia was manually drawn (CtAnalyser v 1.13.11.0, Bruker) starting from the tibial plateau down to the distal junction with the fibula. After global segmentation, BV was calculated at the start of the experiment, prior to treatment, and at the study end. No differences were observed between groups at the starting point.

### 2.8 Alkaline phosphatase activity

An alkaline phosphatase assay was performed using a fluorometric Alkaline Phosphatase Assay Kit (ab83371, Abcam) following the manufacturer's instructions. Fluorescence (excitation 360 nm, emission 440 nm) was measured in a Spectramax Gemini XS plate reader (Molecular devices). Alkaline phosphatase activity was calculated as the amount of 4‐methylumbelliferone (4‐MU) generated over the reaction time and normalized to the total protein concentration in tissue lysates.

### 2.9 Assessment of tumor necrosis

Upon sacrifice, the hind limb tumor was cut in half along the longitudinal axis, fixed in 4% buffered paraformaldehyde for 2 days and then decalcified for 2 weeks using Osteosoft (#101728, Merck Millipore). After routine paraffin embedding, sections (3–5 µm thick) were stained with hematoxylin and eosin. The amount of tumor necrosis was scored by a veterinary pathologist in a blinded manner and as follows: grade 0, no necrosis; grade 1, necrosis below 20% of the total tumor surface; grade 2, necrosis greater than 20% but less than 40%; grade 3: necrosis greater than 40% but less than 60%; grade 4: necrosis greater than 60% but less than 80%; grade 5, necrosis greater than 80%.

### 2.10 Statistical analysis

All results are presented as mean ± SEM. Normally distributed data were analyzed using either a one‐way ANOVA including all groups or a two‐way ANOVA when comparing two categorical independent, repeatedly measured variables. Multiple comparisons were corrected using Bonferroni's test. For data with a non‐Gaussian distribution, Kruskal–Wallis followed by Dunn's test for multiple comparisons was used.

In the monotherapy experiment, all therapies were compared with the vehicle group as control and with each other.

In the combination therapy experiment, all therapies were compared with the vehicle group as control and the monotherapies with each other. Furthermore, ZOL monotherapy was compared to the combination therapies, that is, ZOL versus ActRIIA‐mFc + ZOL or ZOL versus FST_ΔHBS_‐hFc + ZOL, and finally both combination therapies were compared with each other.

Differences were regarded as statistically significant when *p* < 0.05, and any difference described between groups is implied statistically significant unless otherwise stated.

## 3 RESULTS

### 3.1 In vitro validation of therapeutics

**Figure 1:**
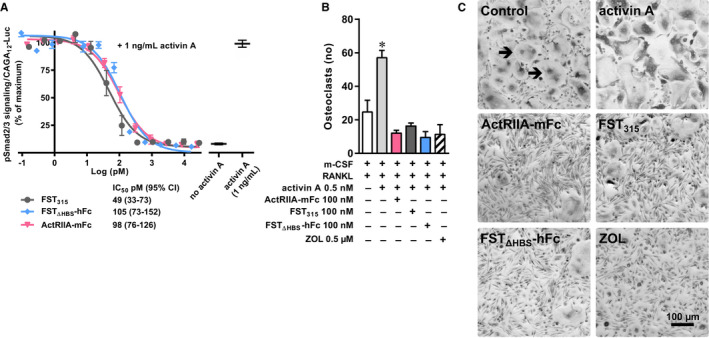
In vitro validation of therapeutics. (A) Ligand neutralization by native FST_315_, FST_ΔHBS_‐mFc, or ActRIIA‐mFc. A luciferase reporter cell line under control of a Smad2/3 sensitive promoter was stimulated with activin A in the presence of different concentrations of native FST_315_, FST_ΔHBS_‐mFc, or ActRIIA‐mFc. Luc, luciferase. (B) CD14‐positive PBMCs were differentiated into TRAP‐positive huge (approximately 100–200 µm), multinucleated osteoclasts in the presence of m‐CSF, RANKL, activin A, and either FST_315_, FST_ΔHBS_‐hFc, ActRIIA‐mFc, or ZOL. Osteoclasts were counted after 14 days of culture. (C) Representative images of TRAP‐positive, multinucleated osteoclasts (arrows) with indicated treatment. One‐way analysis of variance with Bonferroni's correction of multiple comparisons was employed for statistical analysis of the osteoclast count. Data presented as means ± SEM. *p < 0.05 versus control (m‐CSF +RANKL).

The ligand neutralizing activity of ActRIIA‐mFc or FST_ΔHBS_‐mFc was compared to native FST_315_ using an in vitro bioassay. Both ActRIIA‐mFc and FST_ΔHBS_‐mFc were capable of neutralizing activin A with IC_50_'s in the same picomolar range as native FST_315_ (Figure 1A). In addition, the molecules were tested for their ability to block activin A's enhancement of differentiation of human CD14‐positive PBMCs toward multinucleated TRAP positive osteoclasts. The bisphosphonate, ZOL, was included as a control. In the presence of m‐CSF and RANKL, the stimulatory effect of activin A on osteoclastogenesis was abolished by the presence of native FST_315_, ActRIIA‐mFc, FST_ΔHBS_‐mFc, or ZOL alike after 14 days of culture (Figure 1B, C).

### 3.2 Inhibition of the activin receptor signaling pathway in an orthotopic osteosarcoma mouse model

**Figure 2:**
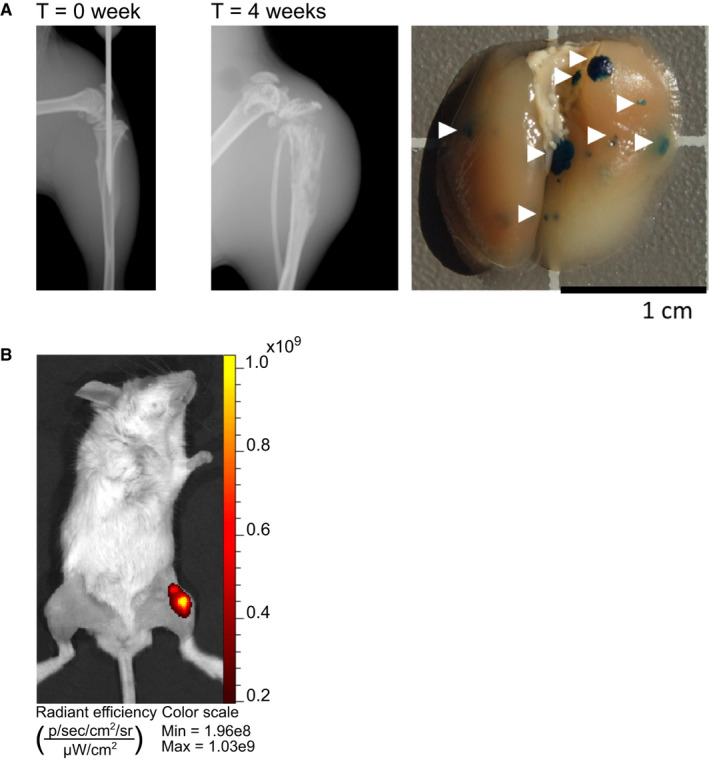
The in vivo intratibial xenograft model of osteosarcoma. (A) Left panel, X‐ray image of the intratibial inoculation of metastatic 143B/*lacZ*/*mCherry* cells into SCID mice at T = 0 week. Middle panel, a representative X‐ray image of visible bone erosions from a specimen in the vehicle‐treated group at the study end, T = 4 weeks. Right panel, a representative example of *lacZ*‐positive pleural metastases (white arrowheads) lungs from a vehicle‐treated specimen at study end. (B) IVIS imaging of *mCherry* fluorescence demonstrating a viable tumor in a vehicle‐treated specimen.

The therapeutic potential of ActRIIA‐mFc or FST_ΔHBS_‐hFc therapy was investigated in a metastatic, orthotopic xenograft osteosarcoma mouse model. Highly metastatic human 143B/*lacZ*/*mCherry* cells were injected into the left tibia of SCID mice. After 5 weeks, vehicle‐treated mice all had macroscopically visible bone erosions and pulmonary metastatic spread similar to the examples shown in Figure 2A. During week 0, IVIS imaging was used to verify a viable tumor in all mice (Figure 2B). Intervention therapy was initiated 11 days after tumor cell injection and is referred to as week 1.

### 3.3 FST_ΔHBS_‐hFc therapy counters weight loss and both ActRIIA‐mFc and FST_ΔHBS_‐hFc reduce osteosarcoma tumor growth

**Figure 3:**
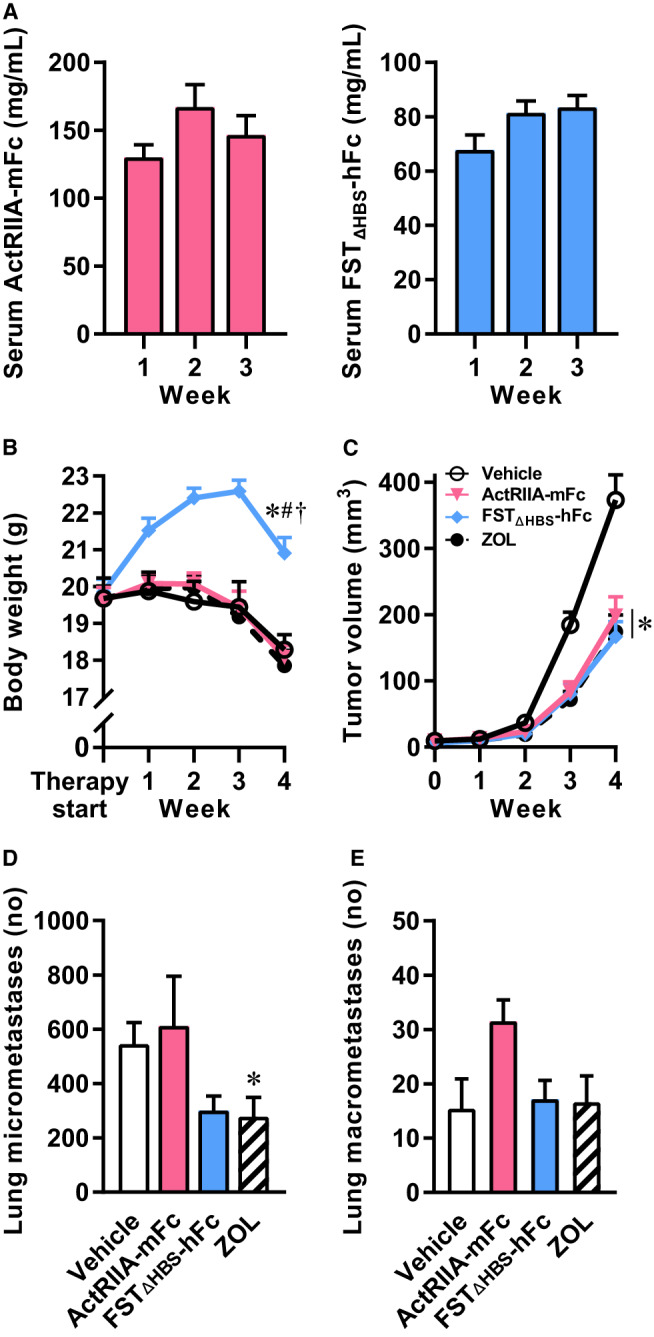
Inhibition of the activin receptor signaling pathway as monotherapy against osteosarcoma. Measurement of ActRIIA‐mFc or FST_ΔHBS_‐hFc in serum 24 h after a single‐dose intravenous injection (A), absolute body weight (B), tumor volume (C), lung micrometastases (D), and lung macrometastases (E) in a monotherapy experiment of IASPs or bisphosphonate in SCID mice. Two‐way analysis of variance with Bonferroni's correction of multiple comparisons was employed for statistical analysis of body weight and tumor volume. Kruskal–Wallis followed by Dunn's multiple comparison was employed for micrometastases. One‐way analysis of variance with Bonferroni's multiple comparison was employed for macrometastases. Data presented as means ± SEM. *p < 0.05 versus vehicle, ^#^ *p* < 0.05 versus ActRIIA‐mFc, ^†^ *p* < 0.05 versus ZOL.

Intervention therapies were administered to the mice twice weekly for 4 weeks. Vehicle, ActRIIA‐mFc (10 mg/kg), and FST_ΔHBS_‐hFc (10 mg/kg) were delivered intraperitoneally, while ZOL (100 µg/kg) was delivered subcutaneously. Serum levels of ActRIIA‐mFc and FST_ΔHBS_‐hFc were measured regularly and demonstrated a high circulating level of each molecule in the bloodstream (Figure 3A).

Following tumor cell injection, mice treated with vehicle, ActRIIA‐mFc, or ZOL gradually developed cachexia (Figure 3B). In contrast, mice treated with FST_ΔHBS_‐hFc increased their body weight regardless of tumor progression until the period between week 3 and 4, where the mice succumbed to the tumor burden. Notwithstanding this final decline, FST_ΔHBS_‐hFc treated mice were 14% heavier than vehicle mice at the study end.

Tumor volume development was mitigated by all three treatments with nearly a halving of the tumor volume at the study end compared to vehicle treatment (Figure 3C). A tendency for a reduction in the number of lung micrometastases was observed with FST_ΔHBS_‐hFc therapy, but neither micro‐ nor macrometastases were significantly affected by ActRIIA‐mFc or FST_ΔHBS_‐hFc therapy (Figure 3D, E). The lungs of ZOL treated mice showed less lung micrometastases.

### 3.4 Combination therapy with ActRIIA‐mFc and ZOL provide the greatest reduction in osteosarcoma tumor volume

A second in vivo study was conducted to see if additional benefit could be gained by combining ActRIIA‐mFc or FST_ΔHBS_‐hFc with ZOL.

**Figure 4:**
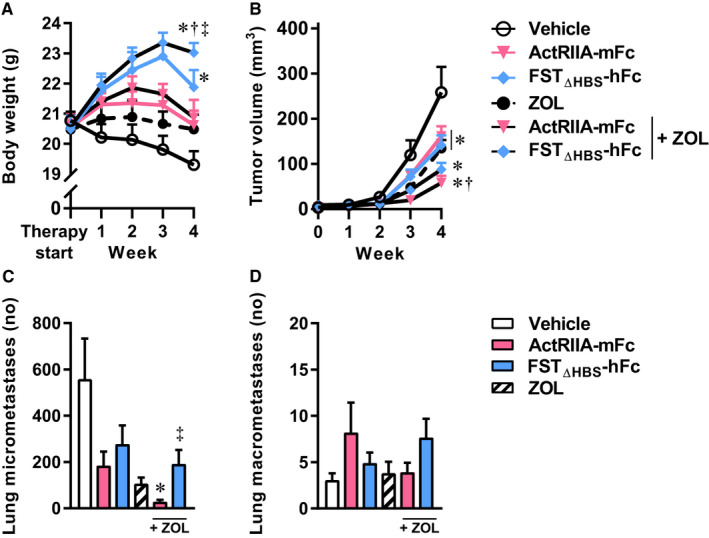
Inhibition of the activin receptor signaling pathway in combination with bisphosphonate therapy against osteosarcoma. Absolute body weight (A), tumor volume (B), lung micrometastases (C), and lung macrometastases (D) in a combination therapy experiment of IASPs and bisphosphonate in SCID mice. Two‐way analysis of variance with Bonferroni's correction of multiple comparisons was employed for statistical analysis of body weight and tumor volume. Kruskal‐Wallis followed by Dunn's multiple comparison was employed for micrometastases. One‐way analysis of variance with Bonferroni's multiple comparison was employed for macrometastases. Data presented as means ± SEM. *p < 0.05 versus vehicle, ^#^ *p* < 0.05 versus ActRIIA‐mFc, ^†^ *p* < 0.05 versus ZOL, ^‡^ *p* < 0.05 versus ZOL + ActRIIA‐mFc.

From week 2 and throughout the study, FST_ΔHBS_‐hFc treated mice were heavier than vehicle‐treated mice (Figure 4A). Neither ActRIIA‐mFc, nor ZOL therapy, nor their combination led to increased body mass at sacrifice, after 4 weeks of treatment.

The capability of ActRIIA‐mFc, FST_ΔHBS_‐hFc, or ZOL to reduce primary tumor development was confirmed (Figure 4B). Tumor volume was further reduced when ActRIIA‐mFc was combined with ZOL (78% reduction vs. vehicle), while FST_ΔHBS_‐hFc in combination with ZOL did not significantly alter tumor volume compared to ZOL therapy alone (Figure 4B).

The combination of ActRIIA‐mFc and ZOL caused a significant reduction in micro‐ but not macrometastases (Figure 4C, D).

In summary, ActRIIA‐mFc and FST_ΔHBS_‐hFc and ZOL were all found to reduce osteosarcoma tumor volume in two independent experiments. FST_ΔHBS_‐hFc countered weight loss, especially in combination with ZOL, and ZOL alone or in combination with ActRIIA‐mFc reduced lung micrometastases.

### 3.5 Both ActRIIA‐mFc and FST_ΔHBS_‐hFc mitigate bone destruction

**Figure 5:**
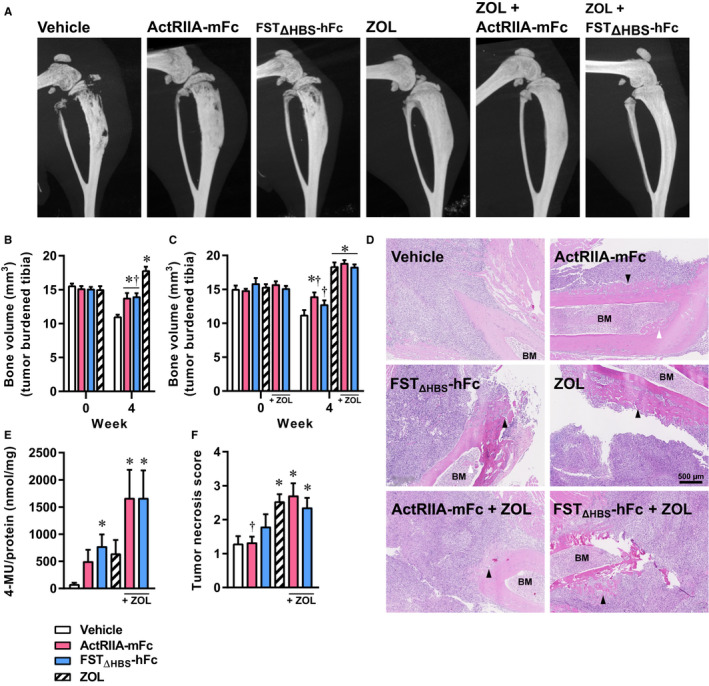
Inhibitors of the activin receptor signaling pathway and bisphosphonate mitigate tumor‐associated bone remodeling. Representative µCT images of tumor bearing hind limbs (A), bone volume of inoculated tibiae in the monotherapy experiment (B), bone volume of inoculated tibiae in the combination therapy experiment (C). In both experiments, the bone loss in the vehicle group between week 0 and 4 was significant, symbols omitted for clarity. Hematoxylin and eosin stained sections indicating periosteal (black arrowhead) and endocortical bone formation (white arrowhead; BM, bone marrow cavity; (D), alkaline phosphatase activity shown as 4‐MU generation per mg of protein in tumor tissue extracts (E), and tumor necrosis score (F). Two‐way analysis of variance with Bonferroni's correction of multiple comparisons was employed for statistical analysis of in vivo μCT data. Kruskal–Wallis followed by Dunn's multiple comparison was employed for the alkaline phosphatase activity. One‐way analysis of variance with Bonferroni's multiple comparison was employed for the tumor necrosis score. Data presented as means ± SEM. *p < 0.05 versus vehicle, ^#^ *p* < 0.05 versus ActRIIA‐mFc, ^†^ *p* < 0.05 versus ZOL, ^‡^ *p* < 0.05 versus ZOL + ActRIIA‐mFc.

We examined the tumor bearing hind limbs in both studies using in vivo µCT. Although tumor‐related osteoid formation does occur in the tibia, intramedullary injection of 143B osteosarcoma cells in SCID mice mainly results in an osteolytic phenotype. The presence of osteolytic lesions was most prominent in the vehicle‐treated group (Figure 5A).

µCT analysis of the tibiae demonstrated ActRIIA‐mFc as well as FST_ΔHBS_‐hFc mitigated the pathological destruction of bone (Figure 5B). This was observable in the combination therapy experiment too, albeit the finding did not reach significance for FST_ΔHBS_‐hFc (Figure 5C). ZOL therapy increased bone mass without any apparent benefit of being combined with ActRIIA‐mFc or FST_ΔHBS_‐hFc (Figure 5C).

Microscopically, localized or multifocal new bone formation was found at the periosteal and to a lesser extent at the endocortical surface in all compound treated groups (Figure 5D). To confirm the anabolic activity in the tumor bone microenvironment, alkaline phosphatase activity was determined in tissue extracts of tumor bearing hind limbs. Alkaline phosphatase is an important indicator of bone mineralization and a prominent increase was observed when ActRIIA‐mFc or FST_ΔHBS_‐hFc was used in combination with ZOL (Figure 5E).

An investigation conducted by a veterinary pathologist (GP) concluded that the primary tumor consisted of large areas of bone and soft tissue effacement associated with infiltrating neoplastic osteosarcoma cells. These cells exhibited mitotic figures, prominent anisocytosis, cellular atypia, and a variable amount of necrosis represented by either multifocal small groups of necrotic neoplastic cells, or more discrete, confluent areas of necrosis infiltrated by neutrophils. Only therapies including ZOL were characterized by a higher necrosis score within the primary tumor compared to the vehicle group, indicating a cytotoxic effect of ZOL (Figure 5F).

Taken together, these results demonstrate that ActRIIA‐mFc and FST_ΔHBS_‐hFc both mitigated osteosarcoma‐induced pathological bone remodeling, albeit to a lesser extent than ZOL (100 µg/kg) with the applied dosage regimen of 10 mg/kg, twice weekly. The prominent alkaline phosphatase activity upon add‐on of ZOL to either ActRIIA‐mFc or FST_ΔHBS_‐hFc points toward an increased bone anabolic drive on top of the osteoclastic inhibition and cytotoxicity of ZOL therapy alone.

## 4 DISCUSSION

Osteosarcoma is a deadly cancer associated with extended tumor‐associated pathological bone remodeling. There is an unmet need for therapies that effectively reduce mortality and comorbidity.

In the present study, we explored the use of two different IASPs, a soluble activin type IIA decoy receptor and a follistatin analog, as therapeutics against osteosarcoma in an in vivo intratibial xenograft model.^27^ Two experiments were conducted: An exploratory monotherapy study and a second experiment investigating the use of combination therapy with the established antiresorptive, bisphosphonate. Both IASPs were found capable of reducing tumor growth and tumor‐associated bone remodeling. Furthermore, ActRIIA‐mFc reduced lung metastatic tendency when combined with bisphosphonate and FST_ΔHBS_‐hFc counteracted cachexia.

Previously, ActRIIA‐Fc therapy in murine models of multiple myeloma or metastatic breast cancer has shown inhibition of tumor growth and a reduction in the number of metastases and osteolytic lesions.^13^, ^14^ The data presented here extend these findings and show that ActRIIA‐mFc or FST_ΔHBS_‐hFc therapy may also blunt osteosarcoma primary tumor volume, possibly as a consequence of modulated tumor‐associated bone remodeling: Both ActRIIA‐mFc and FST_ΔHBS_‐hFc were capable of abolishing activin A's enhancement of osteoclastogenesis. In turn, this could lead to a reduction in the release of growth factors, thus, breaking the “seed and soil” cycle by denying implantation or growth of tumor cells.

In relation to chemotherapy, the reduction in primary tumor volume produced by the combination of a bisphosphonate and ActRIIA‐mFc was roughly equivalent to high‐dose, intra‐arterial Cisplatin therapy in a study employing the same model of osteosarcoma.^30^ Still, it should be emphasized that these are two separately performed studies and therefore not directly comparable.

In many bone‐associated cancers, bisphosphonates such as zoledronic acid are considered standard of care therapy against skeletal‐related events and preclinical studies have reported its benefit in treatment of osteosarcoma too.^20^, ^21^ Bisphosphonates are believed to be directly cytotoxic to a number of tumor cell types,^31^ which is consistent with our finding of an increased tumor necrosis score in the bisphosphonate‐treated groups. In contrast, neither ActRIIA‐mFc nor FST_ΔHBS_‐hFc had direct cytotoxic effects. However, the use of bisphosphonate in osteosarcoma treatment has not been implemented despite its proposed antitumor effect. This is because clinical trials have yet to demonstrate its merit against osteosarcoma, representing a discrepancy between preclinical and clinical results.^32^

We were surprised to find that alkaline phosphatase activity was enhanced in tumor extracts when either IASP was combined with bisphosphonate. The clinical implication of this is difficult to gauge, but the phenomenon warrants further investigation, especially since the observed effect was virtually additive.

A third TGFβ superfamily member, myostatin, also signals through activin type II receptors and is also neutralized by IASPs (most effectively by FST). In mammals, myostatin is renowned as a negative regulator of skeletal muscle mass, although in primates it appears to share this feature with activin A.^33^ Body weight and muscle mass deterioration often follow in the wake of cancer and there is a vacuum of therapies against this condition known as cachexia.^34^, ^35^, ^36^, ^37^, ^38^ Cachexia leads to a reduction in performance status and quality of life and signifies the terminal period of the patient.^39^ The ability of FST_ΔHBS_‐hFc to increase body weight in the face of progressing osteosarcoma is, therefore, an interesting feature and it is likely attributable to skeletal muscle preservation as has been noted in other cancer types with some IASPs.^40^, ^41^ ActRIIA‐mFc did not significantly alter body weight and this difference between the IASPs may likely be attributed to ActRIIA‐mFc not blocking myostatin as strongly as FST_ΔHBS_‐hFc.^16^

In conclusion, ActRIIA‐mFc and FST_ΔHBS_‐hFc therapy both showed a potential for reducing primary tumor volume and tumor‐associated bone remodeling alone or in combination with bisphosphonate. Moreover, FST_ΔHBS_‐hFc was found to counter cachexia and ActRIIA‐mFc reduced the number of lung metastases when combined with bisphosphonate. This is the first study demonstrating that in vivo inhibition of the activin receptor signaling pathway may be utilized as an intervention against osteosarcoma.

Within the last year, the first IASP, an ActRIIB based molecule, has received FDA approval for use in transfusion dependent beta thalassemia or lower‐risk myelodysplastic syndromes. Moreover, an ActRIIA‐based molecule has received FDA breakthrough therapy designation for pulmonary arterial hypertension and is currently being investigated in phase 2 (NCT03738150). The findings of the present study encourage further investigation of this drug class in osteosarcoma treatment too.

## AUTHOR CONTRIBUTIONS

Concept and design: DM, ME, SMB; acquisition of data: DM, AG, GP, ON, ME, SMB; analysis and interpretation of data: DM, AL, ME, SMB; drafting/revising the manuscript: DM, AL, WB, BF, ME, SMB. All authors have read and approved the final version of the manuscript and agreed to be accountable for all aspects of the work.

## ETHICAL APPROVAL

In vivo experiments were conducted with the approval of the Veterinary Office Kanton Zurich, Switzerland (animal application license 42/2013) and in accordance with the guidelines of the Swiss Federal Veterinary Office.

## NCT NUMBERS

A clinical trial was referenced in the discussion (NCT03738150). The authors have no affiliation with this trial.

## Data Availability

The data that support the findings of this study are not publicly available but may be made available by the corresponding author upon reasonable request.
